# Muscle Strength and Male Sexual Function

**DOI:** 10.3390/jcm13020426

**Published:** 2024-01-12

**Authors:** Anders Flataker Viken, Silver Peeter Siiak, Vivi Schlünssen, Elin Helga Thorarinsdottir, Svein Magne Skulstad, Sanjay Gyawali, Randi Jacobsen Bertelsen, Francisco Gómez Real

**Affiliations:** 1 Department of Clinical Science, University of Bergen, 5021 Bergen, Norway; randi.j.bertelsen@uib.no (R.J.B.); francisco.real@uib.no (F.G.R.); 2 Tartu University Andrology Centre, 50406 Tartu, Estonia; silverpeeter.siiak@kliinikum.ee; 3 Research Unit for Environment, Occupation and Health, Danish Ramazzini Centre, Aarhus University, 8000 Aarhus, Denmark; vs@ph.au.dk; 4 Department of Public Health, Aarhus University, 8000 Aarhus, Denmark; 5 Primary Health Care of the Capital Area, 103 Reykjavik, Iceland; 6 Department of Occupational Medicine, Haukeland University Hospital, 5021 Bergen, Norwaysanjay.gyawali@ahus.no (S.G.); 7 Department of Gynecology and Obstetrics, Haukeland University Hospital, 5021 Bergen, Norway

**Keywords:** erectile function, erectile dysfunction, male sexual function, muscle, muscle strength, resistance training, exercise, cardiovascular disease, endothelial dysfunction, Aging Males’ Symptoms Scale (AMS)

## Abstract

Sexual dysfunction, in particular erectile dysfunction, is a common complaint among aging men. Obesity, diabetes, hypertension, and smoking are shown to be independent risk factors for erectile dysfunction, while cardiorespiratory fitness is shown to be protective. Less is known about the role of muscle strength in male sexual function. Our objective was to study the association between male sexual function and typical cardiovascular risk factors, together with exercise and muscle strength. We included data from the fourth wave of the RHINE study. Data on anthropometrics, exercise habits, diseases, muscle strength, and sexual function were collected using questionnaires, including the Aging Males’ Symptoms (AMS) scale. We used multivariable logistic regression analysis to measure the association between sexual function and body mass index (BMI), age, smoking, diabetes, hypertension, exercise and muscle strength status. We included 2116 men aged 48–75 from four Nordic-Baltic countries. BMI, age, smoking, diabetes, and hypertension were found to be associated with higher odds of reporting decreased sexual function, while reporting intact muscle strength was associated with lower odds. In a large Nordic-Baltic male study population, we show that known cardiovascular risk factors are associated with decreased sexual function, while reporting intact muscle strength is associated with lower odds of reporting decreased sexual function.

## 1. Introduction

Men at age 55 can expect another 15 years of being sexually active. Men in good health can add 5–7 years to that number [1]. The ability to engage in sexual activity throughout life is a topic that concerns many men, and sexual function, in particular erectile function, have been shown to significantly impact male psychosocial health [2,3,4]. Normal sexual function is characterized as the ability to achieve sexual desire, arousal, and orgasm, and relies on intricate communication between the central, spinal, and peripheral nervous system. The interplay between brain regions, including the cerebral cortex, the limbic system and descending and ascending spinal pathways is central in initiating sexual desire and erection [5].

Male erectile physiology is a complex physiological process. A sequence of coordinated events involving the central nervous system, spinal reflexes and efferent parasympathetic nerve fibers, cavernous endothelial and smooth muscle cells allow for vasodilation and increased blood flow to the corpora cavernosa of the penis [6]. The gaseous messenger molecule nitric oxide (NO), produced by the enzyme nitric oxide synthase (NOS), is the main mediator of penile vasodilation. With sexual arousal and stimulation, cholinergic parasympathetic nerves that innervate the erectile tissue are activated, causing release of both NO and acetylcholine from its nerve terminals. Following cholinergic signal transduction events, endothelial NOS is activated, causing synthesis of endothelial NO [7]. The rising NO levels induce cyclic GMP signaling pathways in the smooth muscle cells, catalyzing events leading to smooth muscle relaxation, dilation of arterioles and increased blood supply. The incoming blood is trapped by expanding sinusoids and venous outflow reduced by compression of penile venular plexuses [6,7]. Any impairments in trapping and confining blood in the penis may lead to erectile dysfunction (ED), which is defined as the failure to attain or maintain persistent penile erection sufficient to perform normal and satisfactory sexual activity [6,8].

ED is the most common sexual problem in men. It is a multifaceted complaint, and individual etiology may exist. However, vasculogenic ED appears to be by far the most common etiology, structuring around dysfunctions of local endothelium and smooth muscles, and tissue remodeling [6]. The presence of ED has been suggested to represent a peek into the health of the cardiovascular system and a prelude of future cardiac events, since ED is highly prevalent in cardiovascular disease (CVD) and the two disorders share several risk factors, including hypertension, diabetes, obesity and aging [9]. A recent study found that the atherosclerotic cardiovascular disease risk score can be used to reliably predict ED [10]. Several of the CVD and ED risk factors are also part of the metabolic syndrome, a cluster of conditions that increase risk of developing CVD. Most patients have dyslipidemia and insulin resistance, and are older, sedentary and obese.

Obesity is found to be associated with erectile and sexual function in several studies [11,12,13,14,15,16,17]. Patients followed up after bariatric surgery show improvements in general sexual function [18], erectile function and parameters of relevance to ED and metabolic syndrome, including fasting blood glucose [15], endothelial function and intima-media thickness of carotid and cavernosal arteries [16], increased levels of follicle-stimulating hormone (FSH) and total and free testosterone (T) [19]. Plasma free and total T levels are shown to be inversely related to obesity [20,21], and obese men with advanced age not infrequently present with symptoms consistent with androgen deficiency or low T levels (late-onset hypogonadism), including ED, loss of libido and muscle mass [22].

Cardiorespiratory fitness offers significant protection against ED [23], and several reviews have concluded on protective effects of aerobic exercise [24,25,26]. The benefits of physical activity on vascular health and CVD are well supported in the medical literature [12,27]. Physical activity is shown to improve endothelial function and endothelium-derived vasodilation [28,29] mobilize endothelial progenitor cells [30], increase bioavailability of NO [31] and improve metabolic health and inflammation [32].

Less is known about how muscle strength affects ED and sexual function in men. An association between sarcopenia (i.e., low muscle mass and strength) and ED have been shown in elderly Turkish and Korean men [33,34], and a parallel decrease in muscle strength and erectile function has been shown in men with type 2 diabetes [35]. Furthermore, muscle mass is found to be preventative of diabetes type 2 and prediabetic states [36,37,38]. In a 10-year follow-up study, muscle mass showed a significant inverse association with CVD incidence risk after adjusting for confounders, implicating muscle mass as cardioprotective [39]. If muscle mass and strength protect male sexual function during aging, this could be an important adjunct to the clinical management of patients suffering from sexual dysfunction, including ED.

In this study we explore risk and protective factors of decreased sexual function in middle-aged and older males from study centers in Norway, Denmark, Iceland, and Estonia. This study explores several facets of male sexual function. Through the Aging Males’ Symptoms scale (AMS) we investigated parameters relevant to ED through self-reported decrease in sexual performance and number of morning erections. Additionally, we address sexual desire/libido, defined as sexual behavior, drive and interest [5].

We hypothesize that Body Mass Index (BMI), age, smoking, hypertension and diabetes are significant risk factors for reporting decreased sexual function. We also hypothesize that meeting recommended levels of weekly exercise and having intact muscle strength protect against reporting decreased sexual function.

## 2. Materials and Methods

### 2.1. Population

Data were collected as part of the Respiratory Health in Northern Europe (RHINE) study. RHINE is a follow-up study of participants located in Nordic-Baltic countries, constituting men and women from Denmark (Aarhus), Norway (Bergen), Iceland (Reykjavik), Sweden (Gothenburg, Uppsala, Umea) and Estonia (Tartu). For this paper we collected data from a RHINE subpopulation who completed The Aging Males’ Symptoms (AMS) scale in the years 2020–2022, comprising middle-aged and adult men from four study centers (Bergen (Norway), Aarhus (Denmark), Reykjavik (Iceland), Tartu (Estonia)).

### 2.2. Data Collection

#### 2.2.1. Questionnaires

**Figure A1:**
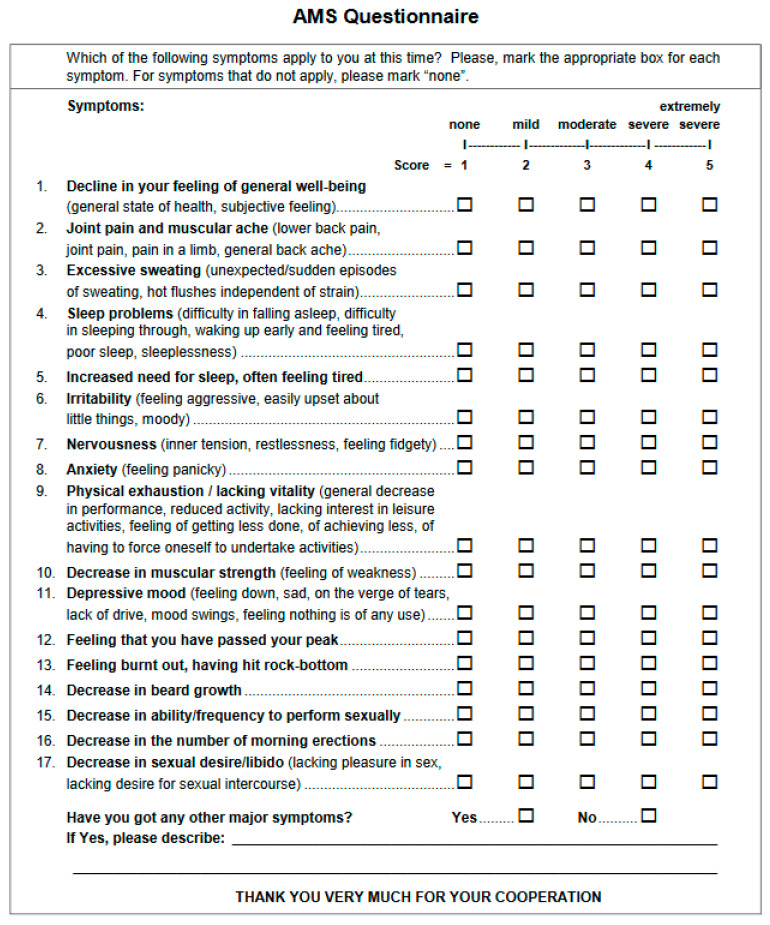
The Aging Males’ Symptom scale (AMS) English version. The ASM scale was developed in Germany in 1999 and has been translated to 12 languages [40].

The main study questionnaire included questions on general characteristics, lifestyle, exercise habits, diseases and medical history, and questions on anthropometric measures including weight and height. The AMS scale was used to assess health parameters specific to the aging male. The scale was originally developed in Germany in 1999 and was designed to examine disease-independent symptoms of males during aging, based on the presumption that males undergo physiological changes leading to complaints like those experienced by females during menopause [40]. A more detailed description of the AMS scale can be found elsewhere [41]. The AMS scale was translated into the language spoken in the country of each study centre. The English version of the scale can be found in Appendix A (Figure A1).

#### 2.2.2. Sexual Function Parameters, Muscle Strength and Exercise

**Figure A2:**
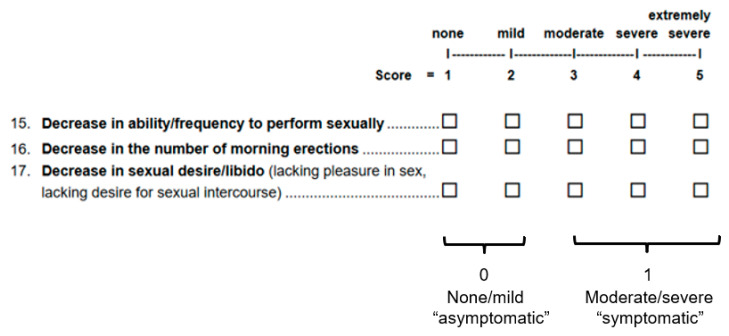
Dichotomization of questions 15, 16 and 17 of the sexual subscale of the Aging Males’ Symptom scale (AMS).

Through the AMS scale we were provided answers to questions relevant to sexual function and muscle strength. The participants were asked whether they suffered “decrease in the number of morning erections”, “decrease in ability/frequency to perform sexually”, “decrease in sexual desire/libido” and “decrease in muscular strength”. The response categories were 1 (none), 2 (mild), 3 (moderate), 4 (severe) and 5 (extremely severe). Similar to Liu and colleagues [42], responses to the three questions related to sexual function were dichotomized to generate binary response variables. Responses of 1 or 2 were set to 0 (asymptomatic (none/mild)), while responses of 3, 4 or 5 were set to 1 (symptomatic (moderate/severe)) (Figure A2, Appendix B). In a similar fashion, we also generated a fourth binary outcome variable “Two or more sexual symptoms”, based on the sum of the three dichotomized sexual function outcomes (decreased morning erections, sexual performance, sexual desire/libido). A sum of 2 was set as the cut off.

For the question related to decline in muscle strength, we generated a two-level categorical variable with levels “None” and “Mild or worse”, with “None” comprising responses of 1 and “Mild or worse” comprising responses 2, 3, 4, and 5. Furthermore, participants were asked questions on frequency, duration and intensity of exercise during the week. Based on their responses to these questions, we were able to assess whether they commit to Worlds Health Organization (WHO)’s recommendations of moderately intense exercise at least three times per week [43], and allocate them accordingly in a categorical variable with levels yes and no. Finally, the participants were asked to rate their own health and based on their responses categorized as “Very good/excellent”, “Good” or “Fair/poor”.

### 2.3. Statistical Analyses

Population descriptive statistics were constructed using median with minimum and maximum values for continuous variables and percentages for categorical variables. Descriptive statistics were conducted for the total population, and for each study centre and each outcome variable. Differences in median values between groups were compared using Kruskal–Wallis rank sum test or Wilcoxon rank sum test, while associations between categorical variables were assessed using Pearson’s Chi-squared test.

Multivariable logistic regression analysis was used to measure the association between variables of interest and self-reported decline in sexual function. To accomplish this, we regressed the dichotomized outcome variables on a set of independent variables of interest. These comprised Body Mass Index (BMI (kg·m^−^^2^)), age, smoking status, hypertension and diabetes, exercise—and muscle strength status, and study centre.

Subsequently, we conducted stratified, subgroup analyses on the study population by levels of self-reported loss of muscle strength. This allowed us to inspect if self-reported muscle strength modified the association between each risk factor and decreased sexual function. Model outputs are presented as adjusted odds ratios (ORs) with corresponding 95% confidence intervals (95% CI) and *p*-values.

R-studio version 4.2.3 was used for analyses and generating tables and figures. A *p*-value below 0.05 was considered statistically significant.

## 3. Results

**Figure 1:**
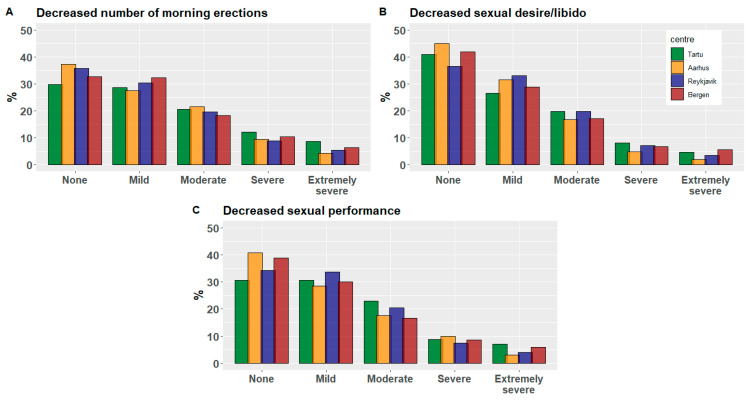
Distribution of severity scores for decreased number of morning erections (**A**), decreased sexual desire/libido (**B**) and decreased sexual performance (**C**) across study centers Tartu, Aarhus, Reykjavik and Bergen.

We included 2116 male subjects from four study centers across four Nordic-Baltic countries. Only participants with complete data for all relevant variables were included. Total and center stratified population characteristics are shown in Table 1. The median population BMI was 27, and the median age was 61 years, with an age range of 48.4 to 74.9. As for smoking status, 12% and 44% were current and ex-smokers, respectively. Tartu showed a markedly higher current smoking prevalence than the other centers. The prevalence of ever being diagnosed with diabetes or hypertension was at 9.1% and 39%, respectively. A slightly higher prevalence of diabetes and hypertension was seen in Reykjavik. Less than 50% reported to adhere to weekly recommended exercise levels, and 60% reported to suffer at least a mild decrease in muscle strength. Reykjavik saw the highest prevalence (72%) of participants reporting suffering at least a mild decrease in muscle strength. For the outcome variables decreased number of morning erections, sexual performance and sexual desire/libido, Tartu saw the highest proportion of participants residing in the category of moderate/severe (symptomatic) (Table 1). This is also apparent in Figure 1, showing the center-specific distribution of severity levels none, mild, moderate, severe and extremely severe for all three sexual function parameters.

**Figure 2:**
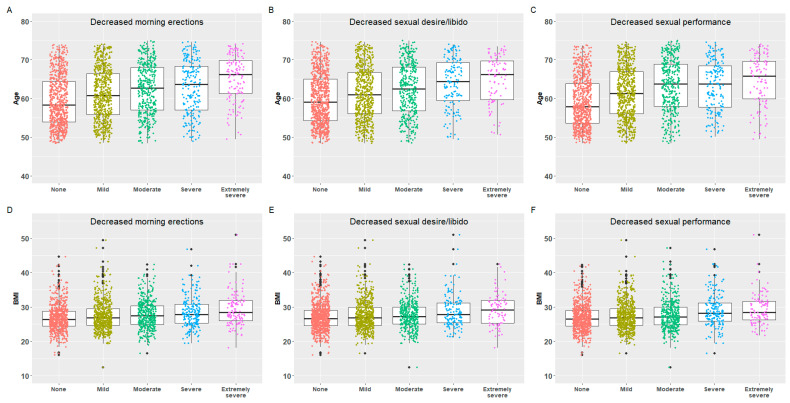
The association between age (**A**–**C**) and BMI (Body Mass Index) (**D**–**F**) and severity scores in three sexual function parameters of the Aging Males’ Symptoms (AMS) scale.

**Figure 3:**
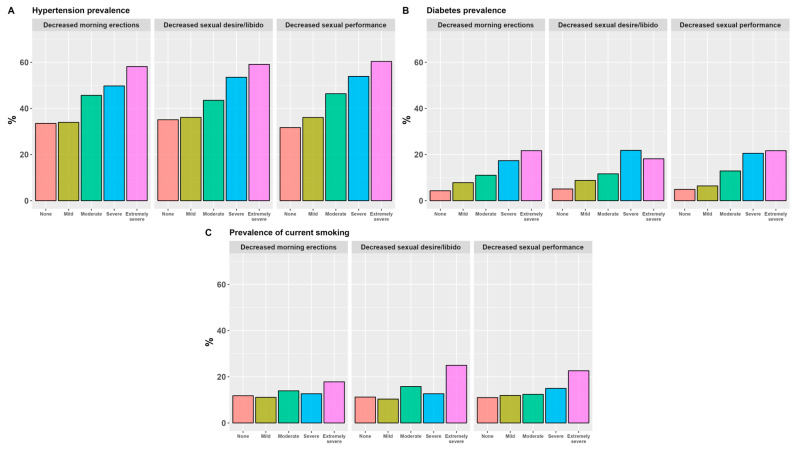
The percentage of reported hypertension (**A**), diabetes (**B**) and current smoking (**C**) on severity scores in three sexual function parameters of the sexual subscale of the Aging Males’ Symptom (AMS) scale.

Across all outcome variables, higher age and BMI was seen for participants residing in the moderate/severe category. Similarly, the moderate/severe category saw a higher prevalence of current and ex-smoking, in addition to ever being diagnosed with diabetes and hypertension. These participants more frequently report not meeting weekly recommended exercise level and more frequently suffer a mild or worse decline in muscle strength (Appendix C, Table A1). A monotonous increase in age, BMI and prevalence of hypertension, diabetes and current smoking was seen for the incremental severity scores for all sexual function outcome variables (Figure 2 and Figure 3).

**Figure 4:**
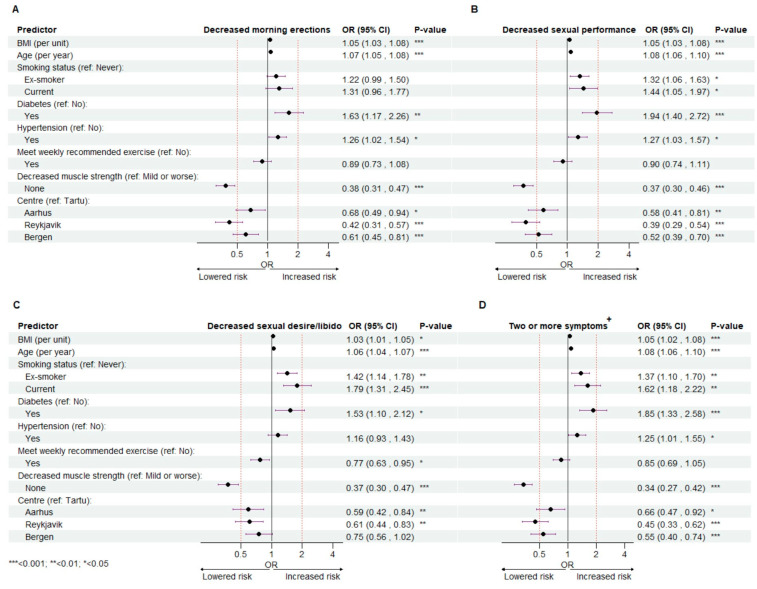
Multivariable logistic regression on the association between predictors of interest and risk of suffering moderate/severe decrease of number of morning erections (**A**), sexual performance (**B**) and sexual desire/libido (**C**), and risk of suffering two or more symptoms of decreased sexual function (**D**). Each outcome level (**A**–**D**) was regressed on independent variables (BMI, age, smoking status, diabetes, hypertension, exercise- and muscle strength status and study centre) using a multivariable logistic regression approach on 2116 male participants. Each forest plot displays the model outcome, with adjusted odds ratios (OR), 95% confidence intervals (CI) and significance level (*p*-values). +: Suffers moderate/severe decrease in two or more symptoms/parameters of sexual function (morning erections; sexual performance; libido/sexual desire).

In Figure 4, forest plots display the outcomes of the modelled association between decreased morning erections (A), sexual performance (B), sexual desire/libido (C), suffering two or more sexual symptoms (D) and BMI, age, smoking status, diabetes, hypertension, exercise level, muscle strength and study center. Associations are shown as adjusted odds ratios (ORs) with corresponding 95% confidence intervals (95% CI) and significance levels (*p*-values).

Every unit increase in BMI was associated with a significantly higher odds of reporting to suffer moderate/severe decrease in number of morning erections (OR 1.05 (1.03–1.08)), sexual performance (OR 1.05 (1.03–1.08)), sexual desire/libido (OR 1.03 (1.01–1.05)), and two or more of these sexual symptoms (OR 1.05 (1.02–1.08)). Findings of similar significance were seen for age, with every additional year increasing the odds of moderate/severe decrease in number of morning erections, sexual performance and -desire/libido (OR 1.07 (1.05–1.08), OR 1.08 (1.06–1.10)), OR 1.06 (1.04–1.07), respectively), and suffering two or more symptoms (OR 1.08 (1.06–1.10)) (Figure 4A–D).

In our study, being diagnosed with diabetes is a consistent risk factor for negative outcome, associated with significant increased odds of reporting moderate/severe decrease in number of morning erections (OR 1.63 (1.17–2.26)), sexual performance (OR 1.94 (1.40–2.72)), sexual desire/libido (OR 1.53 (1.10–2.12)) and suffering two or more symptoms (OR 1.85 (1.33–2.58)) (Figure 4A–D). Receiving a hypertension diagnosis was associated with a significant increased odds of reporting moderate/severe decrease in number of morning erections (OR 1.26 (1.02–1.54)), sexual performance (OR 1.27 (1.03–1.57)) and suffering two or more sexual symptoms (OR 1.25 (1.01–1.55)) (Figure 4A,B,D), but not sexual desire/libido (Figure 4C).

We did not see a significant association between being a former or current smoker and the likelihood of reporting decreased number of morning erections (Figure 4A). In contrast, however, both former and current smoking were significant risk factors for reporting decreased sexual performance (OR 1.32 (1.06–1.63), OR 1.44 (1.05–1.97)), sexual desire/libido (OR 1.42 (1.14–1.78), (OR 1.79 (1.31–2.45)) and suffering two or more symptoms (OR 1.37 (1.10–1.70), (OR 1.62 (1.18–2.22)). Furthermore, in reference to those who never have smoked, being a current smoker appeared to lead to a slightly higher odds of negative outcome than being a former smoker (Figure 4B–D).

As this was a multi-center study across the Nordic-Baltic region, we added study center to our models. Compared to Tartu (reference), odds were lower for Aarhus, Reykjavik and Bergen for decreased number of morning erections (OR 0.68 (0.49–0.94), OR 0.42 (0.31–0.57), OR 0.61 (0.45–0.81)), sexual performance (OR 0.58 (0.41–0.81), OR 0.39 (0.29–0.54), OR 0.52 (0.39–0.70)) and suffering two or more symptoms (OR 0.66 (0.47–0.92), OR 0.45 (0.33–0.62), OR 0.55 (0.40–0.74 (Figure 4A,B,D). As for decreased sexual desire/libido, odds were only significantly lower for Aarhus and Reykjavik (OR 0.59 (0.42–0.84), OR 0.61 (0.44–0.83)), in comparison to Tartu (Figure 4C).

We have inconsistent findings on the association between exercise and sexual function. We found that meeting recommended exercise level was associated with lower odds of reporting decreased sexual desire/libido (OR 0.77 (0.63–0.95)) (Figure 4C), but no significant associations were seen between meeting recommended level of weekly exercise and the other sexual function outcomes (Figure 4A,B,D).

For muscle strength, we consistently show that reporting no loss in muscle strength is associated with a significantly lower odds of reporting to suffer moderate/severe decrease in number of morning erections (OR 0.38 (0.31–0.47)), sexual performance (OR 0.37 (0.30–46)), sexual desire/libido (OR 0.37 (0.30–0.47)) and odds of suffering two or more sexual symptoms (OR 0.34 (0.27–0.42)) (Figure 4A–D). Additional adjustments for decreased beard growth and increased night sweating (hot flushes) did not change the estimates (results not shown).

**Figure 5:**
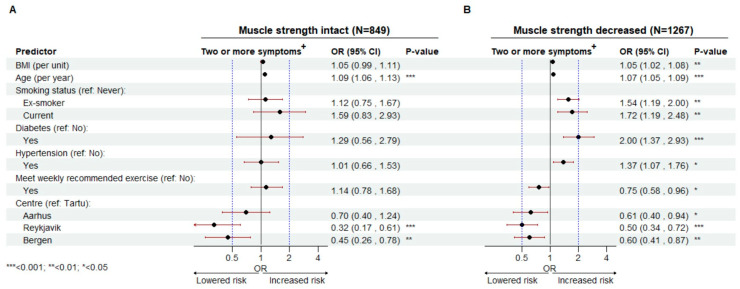
The risk of suffering two or more sexual symptoms regressed on predictors of interest in two different muscle strength subpopulations. The population (N = 2116) was divided by muscle strength (intact (N = 849) (**A**) and decreased muscle strength (N = 1267) (**B**). Multivariable logistic regression was performed separately for both strata. The outcome is presented as adjusted odds ratios (OR) with belonging 95% confidence intervals (CI) and significance level (*p*-values). +: Suffers moderate/severe decrease in two or more symptoms/parameters of sexual function (number of morning erections; sexual performance; libido/sexual desire).

**Figure A3:**
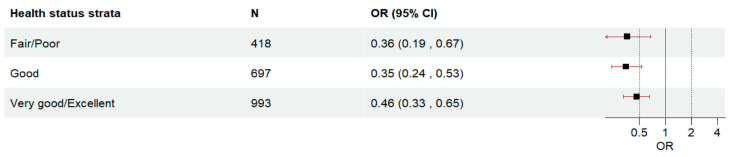
Self-reported intact muscle strength is associated with lower risk of reporting two or more sexual symptoms across three different health status subpopulations. The total population (N = 2116) was divided into three subpopulations/health status strata (Fair/Poor (N = 418); Good (N = 697); Very good/Excellent (N = 993) before multivariable logistic regression was performed separately for all strata to investigate the association between suffering two or more sexual symptoms and BMI, age, smoking status, diabetes, hypertension, recommended weekly exercise, decreased muscle strength and study centre. The figure displays the adjusted odds ratio for decreased muscle strength (None) pulled from the stratified models. The outcome is shown as odds ratios (OR) with 95% confidence intervals (CI).

Subsequently, we divided the total population by muscle strength and conducted stratified regression analyses. In the strata reporting, no decrease in muscle strength, BMI, former and current smoking, diabetes and hypertension are no longer significant predictors of reporting two or more sexual symptoms (Figure 5A). This contrasts with the strata reporting decreased muscle strength, where BMI, former and current smoking, diabetes and hypertension are significant risk factors. Meeting recommended weekly exercise is associated with significantly lower odds, but only in the decreased muscle strength strata (OR 0.75 (0.58–0.96)) (Figure 5B). Age, however, is still a significant risk factor in both strata, with every additional year lived associated with increased odds of negative outcome (Figure 5A,B). Finally, we conducted supplementary subgroup analyses where we stratified the population by their self-reported health. In Figure A3 (Appendix D) we have isolated the model coefficient for decreased muscle strength from all three stratified models. We show that reporting to suffer no decrease in muscle strength remains statistically significant across three different levels of self-reported health.

## 4. Discussion

In this paper, we analyzed AMS questionnaire responses from 2116 middle-aged and older men from four study centers in Bergen, Aarhus, Reykjavik and Tartu, and used multivariable logistic regression to explore how the likelihood of suffering decreased sexual performance, number of morning erections, sexual desire/libido, and two or more of these symptoms depend on various modifiable and non-modifiable factors. We show that increasing BMI and age, diabetes, hypertension and being a former or current smoker are significant risk factors for reporting decreased sexual function. Age, BMI and diabetes were significant risk factors across all studied outcomes of sexual function. Hypertension and being a former or current smoker were significant risk factors in three out of four sexual function outcomes (Figure 4). Reporting intact muscle strength was associated with lower odds of reporting decreased sexual function. Subgroup analysis revealed that muscle strength status may modify the effect of established risk factors. The impact of intact muscle strength remained significant across three different levels of self-reported health status.

Our findings appear to align with the literature concerning risk factors for sexual function decline, in particular ED [9,11,12]. ED is not an uncommon complaint among aging men, with prevalence increasing with age. Any process that impairs the integration of neural or vascular pathways in the penile tissue may lead to ED, but vasculogenic causes are most common. The pathology in vasculogenic ED is multifaceted, but several studies suggest increased systemic inflammatory signaling and endothelium dysfunction to be central [6]. Endothelium dysfunction is a broad term used to characterize the cellular mechanisms driving diseases to the vascular endothelium. The dysfunction is complex but involves the endothelium displaying a phenotype characterized by disturbed NO-cGMP signaling, decreased expression of eNOS, increased production of asymmetric dimethylarginine (competitive eNOS inhibitor), increased production of reactive oxygen species (ROS) and markers of inflammation and cell-adhesion (IL-6, CRP, TNF-alpha, E-selectin, ICAM) and dysregulation of fibrinolytic factors. Systemic effects include increased vascular stiffness and tone, decreased endothelial dependent dilation, immune infiltration and atherosclerosis [44]. In ED, disrupted NO-cGMP pathways and cavernous endothelial dysfunction are likely central molecular and cellular processes driving the pathology, ultimately leading to structural changes and atherosclerosis of the penile vasculature. Many ED patients display increased levels of circulating inflammatory markers, and conditions associated with inflammation like advanced age, obesity, diabetes, and hypertension are all shown to be independent risk factors for ED [6,9]. It is suggested that ED represents a first subclinical presentation of endothelial dysfunction prior to CVD, therefore coined “a canary in the coal mine” [45]. Furthermore, cigarette smoking is known to induce inflammation and ROS signaling in vascular endothelium and a is risk factor for ED in several studies [9]. In our study, former or current smoking were not associated with a significant increased odds of suffering decreased number of morning erections. On the other hand, former or current smoking were associated with a significant increased odds of decreased sexual performance and decreased sexual desire/libido (Figure 4). We suggest that decreased sexual performance is most suitable for assessing male erectile function decline when using the AMS questionnaire, as the risk profile on sexual performance aligned the most with the current literature concerning ED.

Compared to ED, the pathology and risk factors of decreased male sexual desire/libido are less explored. Decreased sexual desire/libido typically presents as lowered interest in sexual thoughts, fantasies, and activity, and is known to gradually decline with age [5,46]. Accordingly, aging was a significant risk factor for decreased sexual desire/libido in our study. Aging typically causes a decrease in T levels, and advanced age coupled with metabolic syndrome increases the risk of late-onset hypogonadism and its related vascular and metabolic comorbidities, together with loss of libido and erectile function [22,47]. The importance of T in regulating male sexual behavior is well recognized, regulating male sexual behavior and libido at the central level and implicating erectile function peripherally [5,6]. It is suggested that sexual desire/libido is a facet of male sexual dysfunction that is more sensitive to low T and less to comorbidities [46]. In our study, hypertension was not associated with significant increased odds of reporting decreased sexual desire/libido, and although age, BMI, diabetes and smoking were statistically significant negative predictors, BMI and diabetes were slightly weaker predictors than for number of morning erections and sexual performance. This could imply slight differences in pathological mechanisms between these facets of male sexual function.

Furthermore, we assessed how participants self-reported level of muscle strength was associated with their sexual function. Reporting to suffer no loss of muscle strength was independently associated with a significantly lower odds of reporting moderate/severe decrease in number of morning erections, sexual performance, libido/desire and suffering at least two of these symptoms (Figure 4). The muscle has only been explored to a limited extent in relation to male sexual function, but muscle mass and strength have been shown to be significantly associated with ED severity in Turkish and Korean men [33,34]. Furthermore, skeletal muscle mass is inversely associated with cardiometabolic disease [36,38] and CVD incidence risk [39]. However, an understanding of the mechanistic basis of potential protective effects of muscle strength and mass in ED is still warranted. Skeletal muscle comprises around 40% of total body mass. It has a continually high metabolic requirement both in rest and activity and is the largest organ site of insulin-induced glucose uptake. In conditions of hyperglycemia, such as prediabetes and diabetes type 2, micro- and macrovascular complications are common [48]. Skeletal muscle may indirectly protect vascular structures of the erectile tissue through its role as a regulator of glucose and insulin homeostasis and prevent the progression of conditions characterized by dysregulated glucose and nutrients, like diabetes and metabolic syndrome. Androgen levels positively influence muscle strength, muscle mass and male sexual function [47]. We acknowledge that testosterone could confound the observed protective effect of muscle strength had it been included in our analyses. Trying to account for this, we did analyses controlling for beard growth and hot flushes, symptoms that can associate with low T [49,50]. The adjustment did not cause important changes in our model estimates, implying that muscle strength may have beneficial effects on sexual function that are independent of androgen levels.

In the same context, it is believed that skeletal muscle may work as an endocrine organ, secreting myokines that have endocrine effects. Myokines are a range of bioactive molecules secreted by skeletal muscle and have typically been characterized as autocrine and paracrine factors that allow for myofiber adaptations [51]. However, certain myokines are also released into circulation, exerting benefits on organ systems like the brain, adipose tissue, vascular bed, and heart [52]. Myokine responses are mostly studied as a post-exercise phenomenon, where IL-6 released from muscle during strenuous activity have received attention for its proposed benefits on metabolism and systemic inflammation. However, the endocrine muscle is an area of vivid research [53], exploring potential muscle-pancreatic beta cells [54], -cardiomyocytes [55], and -adipocyte [52,56] and -vascular bed [57] crosstalk, implicating diabetes, cardiovascular and metabolic health. Similarly, we cannot exclude the possibility that a muscle secretome includes factors that have direct beneficial effects on vascular endothelial and smooth muscle cells that assist in maintaining cavernous arteriolar function. However, more research is needed in this area, including in vivo replication of experimental studies. We also need to understand if effort to maintain skeletal mass and strength have any unique and independent effects in this context.

Our muscles typically undergo atrophy as we age and with increasing rate per decade after 30 [58]. However, our capacity to increase strength and mass is preserved through life [59,60,61]. It is not unreasonable to assume that study participants reporting intact muscle strength employ an intentional effort towards resistance training and healthy lifestyle to maintain their muscle strength and mass. Similarly, they may be indulged with physical labor or other activities that preserve their muscle strength. It is interesting, however, that we failed to see any consistent effects of meeting WHO recommended weekly exercise levels (i.e., at least 3 times a week with moderate intensity). This was surprising given that physical activity has been shown to benefit inflammation in both healthy people and CVD patients [62,63], and shown to benefit endothelial function [28,29,30,31] and ED [24,26,64,65,66]. Self-reported levels of physical activity may not reliably reflect the true duration and intensity of participants’ exercise.

Our findings on subgroup analyses reveal the presence of differential risk profiles between muscle strength subpopulations. In the intact muscle strength strata, BMI smoking, diabetes and hypertension are not associated with a statistically significant increased odds of reporting two or more sexual symptoms (Figure 5). This implies that maintaining muscle strength may downregulate the negative effect of established risk factors. At the same time, this effect may be a function of a healthy lifestyle that also preserves muscle strength. However, our supporting analyses reveal that intact muscle strength remains significant also in men who consider their health to be very good or excellent (Figure A3), implying that muscle strength is protective independent of health status. The mechanisms explaining this effect may be muscle mass and strength benefits on inflammatory activity, vascular and metabolic health that reduce the negative burden of other risk factors. Similar observations and explanations have been carried out in the CVD research field showing that muscle strength improves control of CDV risk factors [67]. Given the strong association between ED and CVD, similar pathways may be involved. However, more research in this area is needed to understand the role of the muscle in preserving sexual function in men, and how it may control the effect of other risk factors of sexual dysfunction. We hope that our findings motivate further research in this direction.

Our study has some limitations and strengths that need to be discussed. First, as the AMS scale has no direct questions on erectile function, we had to make indirect assessment of this facet of male sexual function. We cannot exclude the possibility of different findings if questions exclusively targeted towards assessing erectile function had been used. Secondly, as a questionnaire-based study, we had no objective clinical assessments of the outcomes or the independent variables. This complicates making firm conclusions and interpretations of biological mechanisms behind our results. Additionally, questionnaires are vulnerable to reporting bias. However, it is reassuring that our findings are in line with the literature assessing risk factors of decreased sexual function in aging men, suggesting that questionnaires can be used to reliably assess the association between comorbidities and sexual function in aging men. Another strength of our study is its multinational and multiregional design. To our knowledge, this is the first study to address sexual function decline in middle-aged and older men across four Nordic-Baltic countries. Furthermore, we add to the limited literature on muscle strength and sexual function, further substantiating muscle strength as protective of male sexual function. Additionally, we are the first to demonstrate that muscle strength may modify the effect of known risk factors of sexual function decline. Considering this, we propose that resistance training to promote and maintain muscle strength and mass should be included in the clinical management of middle-aged and older men suffering from sexual function decline, including ED.

## 5. Conclusions

We found that in a large Nordic-Baltic male study population, cardiovascular risk factors BMI, age, previous and current smoking, diabetes and hypertension are associated with increased odds of reporting decreased sexual function. In addition, we show that reporting intact muscle strength is associated with lower odds of reporting decreased sexual function. We speculate that maintaining muscle strength may assist in preserving metabolic and vascular health that positively influence several facets of male sexual function during aging and propose that resistance training to maintain muscle strength and mass may be an important adjuvant to the clinical management and prevention strategies of male sexual function decline.

## Figures and Tables

**Table 1:** Population characteristics (N = 2116).

	By Study Centre					Total
Characteristic	Tartu N = 369 ^1^	Aarhus N = 396 ^1^	Reykjavik N = 600 ^1^	Bergen N = 751 ^1^	*p*-Value ^2^	N = 2116 ^1^
**Age**	56.1 (48.4, 69.6)	61.1 (50.0, 74.9)	63.1 (50.7, 74.1)	61.5 (49.6, 73.5)	***	61.0 (48.4, 74.9)
**BMI (Body Mass Index)**	27.5 (12.5, 51.0)	26.1 (16.5, 42.4)	27.7 (18.9, 46.8)	26.6 (18.4, 49.4)	***	27.0 (12.5, 51.0)
**Smoking status**					***	
Never	41%	54%	43%	40%		44%
Ex-smoker	38%	37%	47%	48%		44%
Current	21%	9.6%	9.7%	12%		12%
**Diabetes (yes)**	8.1%	6.8%	11%	9.3%		9.1%
**Hypertension (yes)**	39%	36%	44%	37%	*	39%
**Meet recommended weekly exercise (yes)**	34%	42%	45%	54%	***	46%
**Decreased muscle strength**					***	
None	41%	49%	28%	44%		40%
Mild or worse	59%	51%	72%	56%		60%
**Decreased number of morning erections**						
None/mild	59%	65%	66%	65%		64%
Moderate/severe	41%	35%	34%	35%		36%
**Decreased sexual performance**					*	
None/mild	61%	69%	68%	69%		67%
Moderate/severe	39%	31%	32%	31%		33%
**Decreased sexual desire/libido**					*	
None/mild	67%	77%	70%	71%		71%
Moderate/severe	33%	23%	30%	29%		29%
**Two or more sexual symptoms**	38%	31%	33%	30%		33%

^1^ Median (Minimum, Maximum); %. ^2^ Kruskal–Wallis rank sum test; Pearson’s Chi-squared test. *** *p* < 0.001; * *p* < 0.05.

## Data Availability

Datasets are made available upon justifiable request and with the consent of the relevant national ethics committee. Requests are directed to https://rhine.w.uib.no/, accessed on 7 December 2023.

## Appendix C

**Table A1:** Population characteristics by outcome variables.

	Decreased Number of Morning Erections			Decreased Sexual Performance			Decreased Sexual Desire/Libido		Two or More Sexual Symptoms			
**Variable**	**None/Mild**, N = 1358 ^1^	**Moderate/Severe**, N = 758 ^1^	***p*-Value** ^2^	**None/Mild**, N = 1427 ^1^	**Moderate/Severe**, N = 689 ^1^	***p*-Value** ^2^	**None/Mild**, N = 1500 ^1^	**Moderate/Severe**, N = 616 ^1^	***p*-Value** ^2^	**0**, N = 1424 ^1^	**1**, N = 692 ^1^	***p*-Value** ^2^
**Age**	59.7 (48.4, 74.0)	63.5 (48.4, 74.9)	***	59.6 (48.4, 74.5)	64.0 (48.4, 74.9)	***	59.9 (48.4, 74.6)	63.5 (48.5, 74.9)	***	59.6 (48.4, 74.5)	64.0 (48.4, 74.9)	***
**BMI**	26.6 (12.5, 49.4)	27.8 (16.5, 51.0)	***	26.6 (16.1, 49.4)	27.6 (12.5, 51.0)	***	26.8 (16.1, 49.4)	27.7 (12.5, 51.0)	***	26.6 (16.1, 49.4)	27.8 (12.5, 51.0)	***
**Smoking status**			***			***			***			***
Current	11%	14%		11%	15%		11%	16%		11%	15%	
Ex-smoker	41%	49%		41%	51%		41%	51%		41%	51%	
Never	47%	36%		48%	35%		48%	33%		48%	34%	
**Hypertension (yes)**	34%	49%	***	34%	51%	***	36%	48%	***	34%	50%	***
**Diabetes** **(yes)**	6.0%	15%	***	5.6%	16%	***	6.7%	15%	***	5.7%	16%	***
**Meet recommended weekly exercise**	49%	39%	***	49%	39%	***	49%	37%	***	50%	38%	***
**Decreased muscle strength**			***			***			***			***
None	49%	24%		49%	23%		48%	22%		49%	21%	
Mild or worse	51%	76%		51%	77%		52%	78%		51%	79%	

^1^ Median (Minimum, Maximum); %, ^2^ Wilcoxon rank sum test; Pearson’s Chi-squared test, *** <0.001.
